# IV Thrombolysis Facilitates Interventional Reperfusion in Non‐Cardioembolic but Not Cardioembolic Stroke

**DOI:** 10.1002/acn3.70370

**Published:** 2026-03-24

**Authors:** Annahita Sedghi, Daniel P. O. Kaiser, Martin Arndt, Norma J. Diel, Erik Simon, Witold H. Polanski, Volker Puetz, Hagen B. Huttner, Timo Siepmann

**Affiliations:** ^1^ Department of Neurology, Medical Faculty and University Hospital Carl Gustav Carus TUD Dresden University of Technology Dresden Germany; ^2^ Institute of Neuroradiology, Medical Faculty and University Hospital Carl Gustav Carus TUD Dresden University of Technology Dresden Germany; ^3^ Department of Neurosurgery, Medical Faculty and University Hospital Carl Gustav Carus TUD Dresden University of Technology Dresden Germany

**Keywords:** etiology, ischemia, stroke, thrombectomy, thrombolysis

## Abstract

**Objective:**

Intravenous thrombolysis (IVT) before thrombectomy for ischemic stroke may alter clot structure and procedural performance. We investigated how IVT relates to thrombectomy metrics across stroke etiologies.

**Methods:**

We performed a time‐to‐event analysis of consecutive patients with anterior circulation large vessel occlusion (acLVO) stroke from a prospective thrombectomy registry at a German tertiary stroke center (January 2017–January 2023). The associations between IVT and groin‐to‐recanalization time and number of aspiration attempts were assessed using multivariable stratified Cox regression adjusted for demographic, cardiovascular, and stroke‐related variables.

**Results:**

Of 1702 patients screened, 798 (413 female [51.8%], median age 77 years [66, 84; IQR]) underwent thrombectomy. IVT was administered to 395 (49.5%) patients, and successful reperfusion (mTICI ≥ 2b) was achieved in 680 (85.2%) patients. In non‐cardioembolic stroke, IVT facilitated clot removal, yielding a 40% higher likelihood of successful reperfusion at any time point compared with direct thrombectomy (aHR 1.40; 95% CI [1.08, 1.81]; *p* = 0.01) and a 36% reduction of aspiration attempts (IRR = 0.64, 95% CI [0.50–0.84], *p* = 0.001). In cardioembolic stroke, IVT did not alter the incidence of successful reperfusion during thrombectomy (aHR 1.13; 95% CI [0.92, 1.39]; *p* = 0.26) or the number of aspiration attempts (combined IRR ≈ 1.00, 95% CI [0.82–1.22]) but was associated with a 43% lower likelihood of successful reperfusion throughout the intervention if distal thrombus migration occurred (aHR 0.57; 95% CI [0.33, 0.96]; *p* = 0.03).

**Interpretation:**

IVT was associated with faster reperfusion during thrombectomy in non‐cardioembolic acLVO, whereas in cardioembolic stroke with distal clot migration it was associated with delayed reperfusion.

## 1 Introduction

Thrombectomy is the standard treatment for acute ischemic stroke (AIS) with large vessel occlusion (LVO). Intravenous thrombolysis (IVT) is the recommended first‐line therapy within 4.5 h of symptom onset, and beyond this time if there is evidence of salvageable brain tissue on perfusion imaging [1]. However, the use of IVT before thrombectomy is debated, as it increases risk of hemorrhage. Well‐designed studies comparing direct thrombectomy to bridging therapy with IVT show mixed results [2, 3, 4, 5, 6]. A recent Cochrane systematic review and meta‐analysis including six randomized controlled trials of bridging IVT did not demonstrate evidence of a difference in functional outcome, mortality, or intracranial hemorrhage, when compared with direct thrombectomy. The authors emphasized the need for research to identify time‐ and person‐specific factors that influence the effect of IVT among patients undergoing thrombectomy [7]. Variations in the proportions of fibrin and red blood cell (RBC) content are exhibited by clots of patients with LVO, mainly between clots of cardiac and non‐cardiac origin. These variations may influence the response to IVT before thrombectomy [8, 9]. We aimed to assess the association of IVT with the time from groin puncture to recanalization in patients with AIS who underwent thrombectomy for anterior circulation LVO (acLVO) depending on whether the cause was cardioembolic or non‐cardioembolic.

## 2 Patients and Methods

### 2.1 Ethics Statement

Our study was approved by the local institutional review board (Ethikkommission an der TU Dresden, institutional review board reference number: EK 272072017). Written informed consent for participation was waived in accordance with the national legislation and the institutional requirements.

### 2.2 Study Design

We conducted a post hoc analysis of our prospective registry of consecutive thrombectomy‐eligible patients treated from January 1, 2017 to January 1, 2023 at the tertiary stroke center of University Hospital Carl Gustav Carus in Dresden, Germany. Our thrombectomy registry includes both ‘mothership’ patients and those transferred from one of 13 hub‐and‐spoke telestroke network spokes or from one of eight partner primary stroke centers in eastern Saxony as previously described [10]. Parameters not available in our registry were extracted via chart review by an investigator (AS). Stroke etiology was determined according to TOAST criteria by senior stroke consultants during clinical routine and subsequently validated through a secondary central review of all medical records and diagnostic findings by a study investigator (AS). A list of parameters and their modes of acquisition is provided in the Supporting Information (Information S1, Table S1). Results are reported in compliance with the Strengthening the Reporting of Observational Studies in Epidemiology (STROBE) statement [11].

### 2.3 Patients

We included adults who received thrombectomy for acLVO in a time‐to‐event analysis. Eligible occlusion sites comprised the intracranial segment of the internal carotid artery or the M1 and/or M2 segment of the middle cerebral artery. Distal thrombus migration was defined as an anatomically distinct shift of the occlusion site from a proximal to a more distal arterial segment (e.g., from the intracranial ICA to the M1‐segment, or from the M1‐ to the M2‐segments of the MCA). This was adjudicated by comparing the initial CT angiography with the first series of the digital subtraction angiography or, in the case of inter‐hospital transfer, with a repeat CT angiography performed at the tertiary center prior to thrombectomy. Thus, distal thrombus migration was defined as a pre‐procedural event occurring prior to any endovascular maneuver, thereby ensuring that the observed shifts were independent of the subsequent interventional device strategy. Definitions of variables and patient characteristics are detailed in the Supporting Information (Information S1).

### 2.4 Statistical Analysis

Differences between two groups in demographic, clinical, imaging and procedural characteristics were assessed using Fisher's exact test and Chi^2^‐test for binary and categorical data and Mann–Whitney *U*‐test for ordinal and non‐normally distributed continuous data, where appropriate.

For the main analysis, a time‐to‐event analysis was performed using Cox proportional hazards regression analysis. The primary outcome was time from groin puncture to successful angiographic reperfusion. Anterograde reperfusion was quantified by the modified Thrombolysis in Cerebral Infarction (mTICI) score. Cases were classified as failures if mTICI < 2b, indicating incomplete or poor reperfusion. In cases where procedural times were missing because of lack of any recanalization (mTICI 0 or thrombus not reached), we assigned a censoring time of 500 min to represent an upper limit of observed procedural duration as it exceeded the longest observed groin‐to‐recanalization time in our entire cohort (396 min) with buffer. The distribution of groin‐to‐recanalization times is shown in the Supporting Information (Figure S1). Patients with good reperfusion and missing data on procedural times were excluded from the available case analysis. All patients were considered to enter the risk set at time zero (the time of groin puncture), and follow‐up continued until either the event (successful reperfusion) or right censoring. We constructed a multivariable stratified Cox regression model to assess the association between IVT and time to successful reperfusion. The model was adjusted for relevant clinical and procedural variables, including age, baseline stroke severity (NIHSS), presence of a tandem occlusion or carotid T occlusion, vessel occlusion site and occurrence of distal thrombus migration. To explore effect modification by stroke subtype, we conducted separate models for patients with cardioembolic stroke (Trial of Org 10172 in Acute Stroke Treatment, TOAST category 2) and those with non‐cardioembolic etiologies (TOAST categories other than 2). Model estimates were obtained using the Breslow method for handling ties. Wald statistics were used to assess the significance of individual covariates. We performed a sensitivity analysis, repeating the main analysis excluding patients with stroke of undetermined etiology from the non‐cardioembolic stroke group. We performed a sub‐analysis in patients with large atherosclerotic stroke among those with a non‐cardioembolic etiology to exclude undetermined and other known causes of stroke using the same tests as in the main analysis.

**FIGURE 1:**
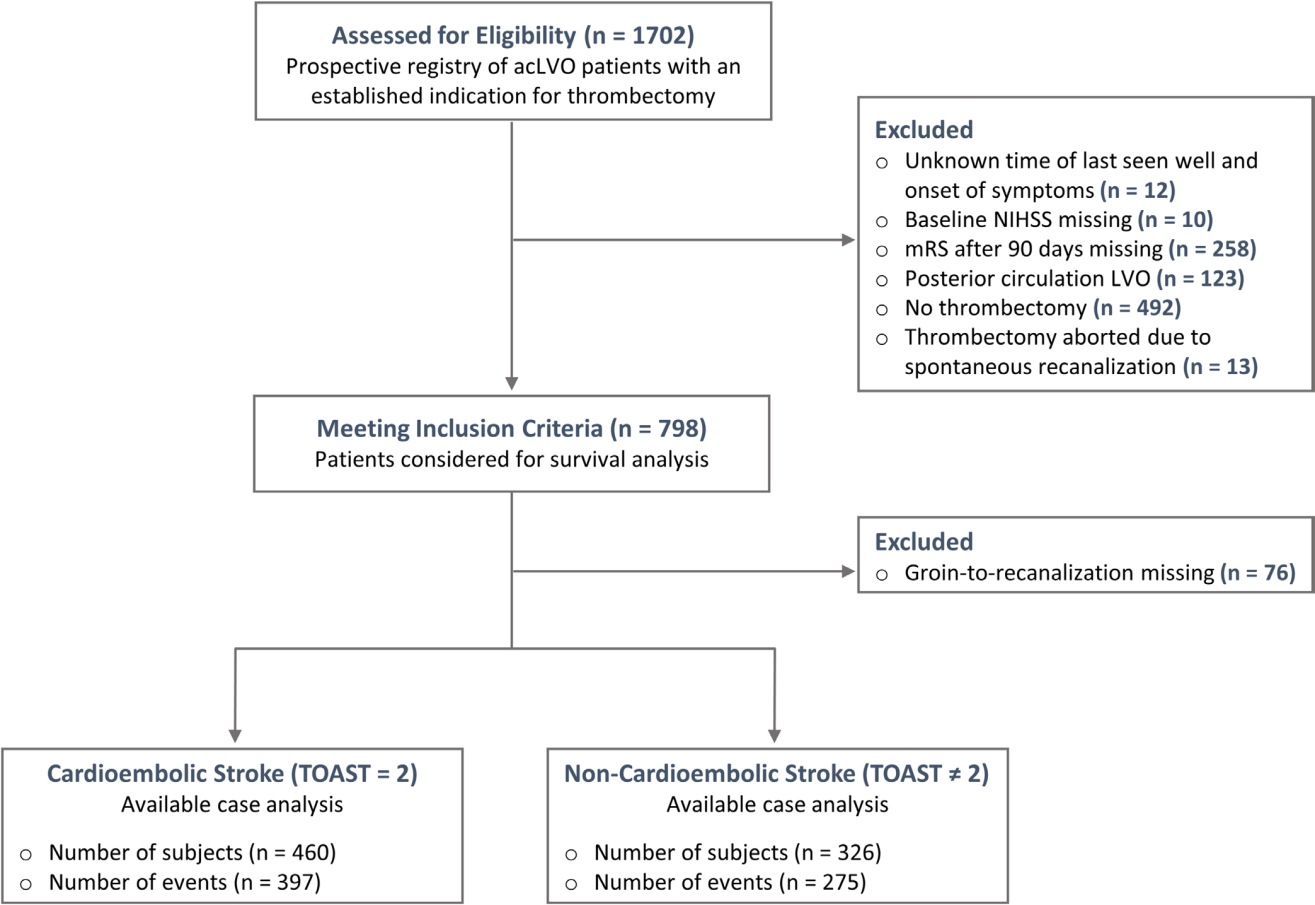
Study flow chart. Flow diagram through the different phases of the study. acLVO, anterior circulation large vessel occlusion; LVO, large vessel occlusion; mRS, modified Rankin scale; NIHSS, National Institutes of Health Stroke Scale; TOAST, Trial of ORG 10172 in Acute Stroke Treatment.

Available case analysis was performed. Statistical significance was defined as a two‐sided alpha (α) level of less than 0.05. All analyses were conducted using Stata (version MP 17.0, StataCorp). Study selection criteria are detailed in the study flowchart (Figure 1).

We assessed the proportional hazards assumption for all covariates using Schoenfeld residuals. Variables showing evidence of non‐proportional hazards were included as stratification factors to allow for separate baseline hazard functions.

Adjusted Kaplan–Meier curves were generated to depict the estimated probability of successful reperfusion over time, comparing patients who did and did not receive bridging IVT. These curves were derived from the Cox proportional hazards model and reflect adjustment for key covariates. This covariate adjustment allows for a more accurate depiction of differences in reperfusion timing attributable to IVT, independent of baseline clinical differences.

The number of aspiration attempts during thrombectomy per patient was analyzed as a count outcome using negative binomial regression to account for overdispersion. Independent variables included IVT pretreatment, stroke etiology according to TOAST criteria, and an interaction term. The selection of covariates was based on clinical relevance and comprised age, ASPECTS, and occlusion site.

The association between groin‐to‐reperfusion (GTR) time per 10 min and functional outcome at 90 days as assessed via the modified Rankin scale (mRS) was investigated using an ordinal logistic regression that was adjusted for clinically relevant patient characteristics, stroke characteristics, interventional parameters, and metabolic markers.

## 3 Results

### 3.1 Patients

We included 798 patients who underwent thrombectomy for AIS due to acLVO (413 females [51.8%], median age 77 years [interquartile range, IQR 66–84 years], baseline NIHSS 15 [IQR, 10–19], median onset‐to‐recanalization time 302 min [IQR, 235–364], median groin‐to‐recanalization time 53 min [IQR, 34–86]). Of those, 395 [49.5%] received IVT prior to thrombectomy. All patients who received intravenous thrombolysis were treated with intravenous alteplase; tenecteplase and intra‐arterial thrombolytics were not used during the study period. Among all patients, successful reperfusion was achieved in 680 patients [85.2%]. Cardioembolic stroke was the underlying cause of stroke in 468 patients [58.9%]. Among patients with non‐cardioembolic stroke, distal thrombus migration occurred in 1/161 (0.6%) patients treated with direct thrombectomy and 14/169 (8.4%) patients receiving bridging IVT, while corresponding rates in cardioembolic stroke were 2/242 (0.8%) and 22/226 (9.7%), respectively (Table 1). Demographic data, vascular risk factors, and clinical and imaging characteristics were balanced between patients who underwent IVT before thrombectomy and those who were treated with direct thrombectomy (Table 1).

**TABLE 1:** Demographic and baseline characteristics.

	Thrombectomy (*n* = 161)	IVT + Thrombectomy (*n* = 169)	*p*	Thrombectomy (*n* = 242)	IVT + Thrombectomy (*n* = 226)	*p*
	Non‐cardioembolic (*n* = 330)			Cardioembolic (*n* = 468)		
*Demographic characteristics*						
Age, years (median [IQR])	73 [64, 80]	68 [59, 78]	0.01	81 [75, 85]	80 [71, 85]	0.09
Sex, female (*n*, %)	65, 40.4	66, 39.1	0.82	138, 57.0	144, 63.7	0.16
Premorbid condition, need assistance (*n*, %)	45, 28.0	26, 15.4	0.007	85, 35.1	65, 28.8	0.17
*Cardiovascular risk factors*						
Arterial hypertension (*n*, %)	139, 86.3	142, 84.5	0.76	223, 92.2	205, 91.1	0.74
Diabetes mellitus (*n*, %)	46, 28.6	44, 26.0		72, 29.8	64, 28.3	
HbA1c, % (median [IQR])	5.8 [5.5, 6.3]	5.7 [5.4, 6.2]	0.38	5.8 [5.4, 6.3]	5.8 [5.5, 6.2]	0.80
LDL‐cholesterol, mg/dL (mean, SD)	2.5, 1.0	2.7, 1.1	0.07	2.1, 0.8	2.3, 0.9	0.27
*Stroke characteristics*						
NIHSS at baseline (median [IQR])	14 [8,18]	15 [11, 19]	0.02	15 [11, 20]	16 [11, 19]	0.52
mRS at 90 days (median [IQR])	4 [2, 6]	2 [1, 4]	0.00	4 [2, 6]	3 [1, 6]	0.0003
ASPECTS (median [IQR])	7 [6, 9]	7 [6, 9]	0.55	7 [6, 9]	8 [6, 9]	0.68
Occlusion side, left (*n*, %)	78, 48.5	92, 54.4	0.27	117, 48.4	119, 52.7	0.36
Occlusion site (*n*, %)			0.15			0.41
ICA intracranial	1, 0.6	4, 2.4	0.37	1, 0.4	3, 1.3	0.36
L, M1 prox.‐distal	131, 81.4	145, 85.8	0.30	192, 79.3	184, 81.4	0.64
M1/2‐transition, M2 prox.‐distal	29, 18.0	19, 11.2	0.09	49, 20.3	39, 17.3	0.48
Carotid T occlusion (*n*, %)	16, 9.9	11, 6.5	0.32	27, 11.2	24, 10.7	0.88
Tandem occlusion (*n*, %)	25, 15.5	46, 27.2	0.01	11, 4.6	20, 8.9	0.07
Leptomeningeal collaterals on DSA (*n*, %)	141, 87.6	135, 81.3	0.13	208, 86.0	182, 80.5	0.14
*TOAST classification (n, %)*						
Large artery atherosclerosis	66, 41.8	81, 47.9	0.27		—	
Cardioembolic	—	—		242, 100	226, 100	
Small vessel occlusion	—	—		—	—	
Stroke of other determined etiology	9, 5.7	13, 7.7	0.51	—	—	
Stroke of undetermined etiology	83, 52.5	75, 44.4	0.15	—	—	
*Interventions (n, %)*						
Drip and ship	103, 64.0	114, 67.5	0.56	148, 61.2	157, 69.5	0.07
Carotid stent (emergency)	38, 23.6	41, 24.5	0.90	—	3, 1.3	0.11
Number of aspirations during thrombectomy (median [IQR])	2 [1, 4]	1 [1, 3]	0.001	2 [1, 3]	2 [1, 3]	0.72
*Sedative regimen (n, %)*			0.09			0.08
Conscious sedation	55, 34.6	42, 25.6		61, 25.3	73, 33.0	
General anesthesia	104, 65.4	122, 74.4		180, 74.7	148, 67.0	
*Procedural times, min (median [IQR])*						
Onset‐to‐needle	—	108 [83, 139]	—	—	105 [80, 135]	—
Onset‐to‐recanalization	329 [259, 447]	303 [232, 365]	0.003	283 [218, 360]	304 [237, 351]	0.34
Groin‐to‐recanalization	66 [34, 108]	53 [36, 86]	0.09	46 [33, 70]	52 [32, 83]	0.25
Needle‐to‐groin	—	128 [55, 158]	—	—	128 [65, 165]	—
*Procedural outcomes (n, %)*						
Distal thrombus migration	1, 0.6	14, 8.4	0.001	2, 0.8	22, 9.7	0.00
mTICI						
0	16, 9.9	18, 10.7	0.86	24, 9.9	12, 5.3	0.08
1	3, 1.9	1, 0.6	0.36	3, 1.2	1, 0.4	0.62
2a	13, 8.1	3, 1.8	0.01	10, 4.1	14, 6.2	0.40
2b	56, 34.8	65, 38.5	0.50	82, 33.9	57, 29.7	0.37
2c, 3	73, 45.3	82, 48.5	0.58	123, 50.8	132, 58.4	0.11
Successful reperfusion (mTICI ≥ 2b)	129, 80.1	147, 87.0	0.10	205, 84.7	199, 88.1	0.35

Abbreviations: ASPECTS, Alberta Stroke Program Early CT Score; ICA, internal carotid artery; IQR, interquartile range; IVT, intravenous thrombolysis; LDL, low density cholesterol; mRS, modified Rankin scale; mTICI, modified treatment in cerebral infarction; NIHSS, National Institutes of Health Stroke Scale; SD, standard deviation; TOAST, Trial of Org 10172 in Acute Stroke Treatment.

A comparison between patients who suffered a non‐cardioembolic versus a cardioembolic stroke is provided in the Supporting Information (Table S2). The amount of missing registry data is low (Table S3).

### 3.2 Effect of IV Thrombolysis on Time to Reperfusion in Non‐Cardioembolic Stroke

**FIGURE 2:**
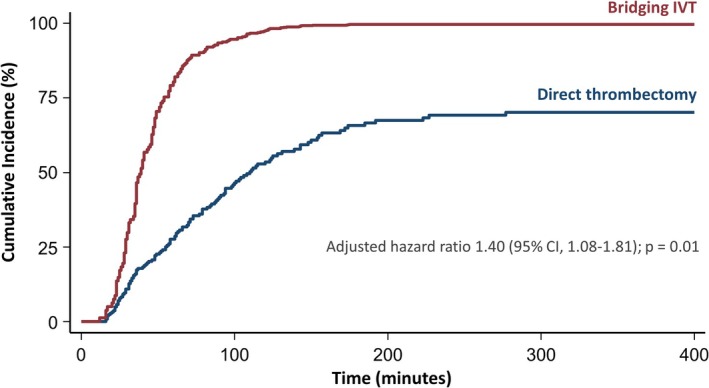
Time to reperfusion during thrombectomy in non‐cardioembolic stroke. Covariate‐adjusted cumulative incidence functions for reperfusion in non‐cardioembolic stroke. The displayed curves represent estimates derived from the multivariable Cox model adjusted for age, distal thrombus migration, National Institutes of Health Stroke Scale at baseline, occlusion site, tandem occlusion, carotid‐T occlusion, carotid stenting, Alberta Stroke Program Early CT Score, leptomeningeal collaterals, sedative regimen. IVT, intravenous thrombolysis.

Successful reperfusion was achieved in 276 (83.6%) patients with non‐cardioembolic stroke. Among patients with non‐cardioembolic stroke, IVT was independently associated with a shorter time to successful reperfusion (Figure 2).

In the stratified Cox proportional hazards model, IVT was associated with an increased hazard of achieving successful reperfusion at any time point during thrombectomy (adjusted hazard ratio [aHR] 1.40, 95% CI [1.08, 1.81], *p* = 0.01). Plausibly, the occurrence of tandem occlusion led to a lower hazard of successful reperfusion at any time point during thrombectomy (aHR 0.70, 95% CI [0.50, 0.98], *p* = 0.036). A detailed description of the Cox proportional hazards model as well as validity assessments is provided in the Supporting Information (Table S4 and Information S2). Stratification by carotid stenting, leptomeningeal collaterals, and sedative regimen was applied to account for potential non‐proportional hazards in these variables. The global and individual test of proportional hazards for each covariate is reported in the Supporting Information (Table S5).

In the sensitivity analysis excluding patients with stroke of undetermined etiology from the group of non‐cardioembolic stroke cases, IVT was associated with an increased hazard of achieving successful reperfusion at any time point during thrombectomy (aHR = 2.06, 95% CI [1.06–3.98], *p* = 0.033). The results of the Cox proportional hazards model, as well as the global and individual proportional hazards tests for each covariate, are reported in the Supporting Information (Tables S6 and S7).

The sub‐analysis in large artery atherosclerotic stroke also showed an independent association between bridging IVT and accelerated thrombectomy with an increased likelihood of successful reperfusion at any given time point during the procedure as detailed in the Supporting Information (Information S3 and Tables S8 and S9).

### 3.3 Effect of IV Thrombolysis on Time to Reperfusion in Cardioembolic Stroke

**FIGURE 3:**
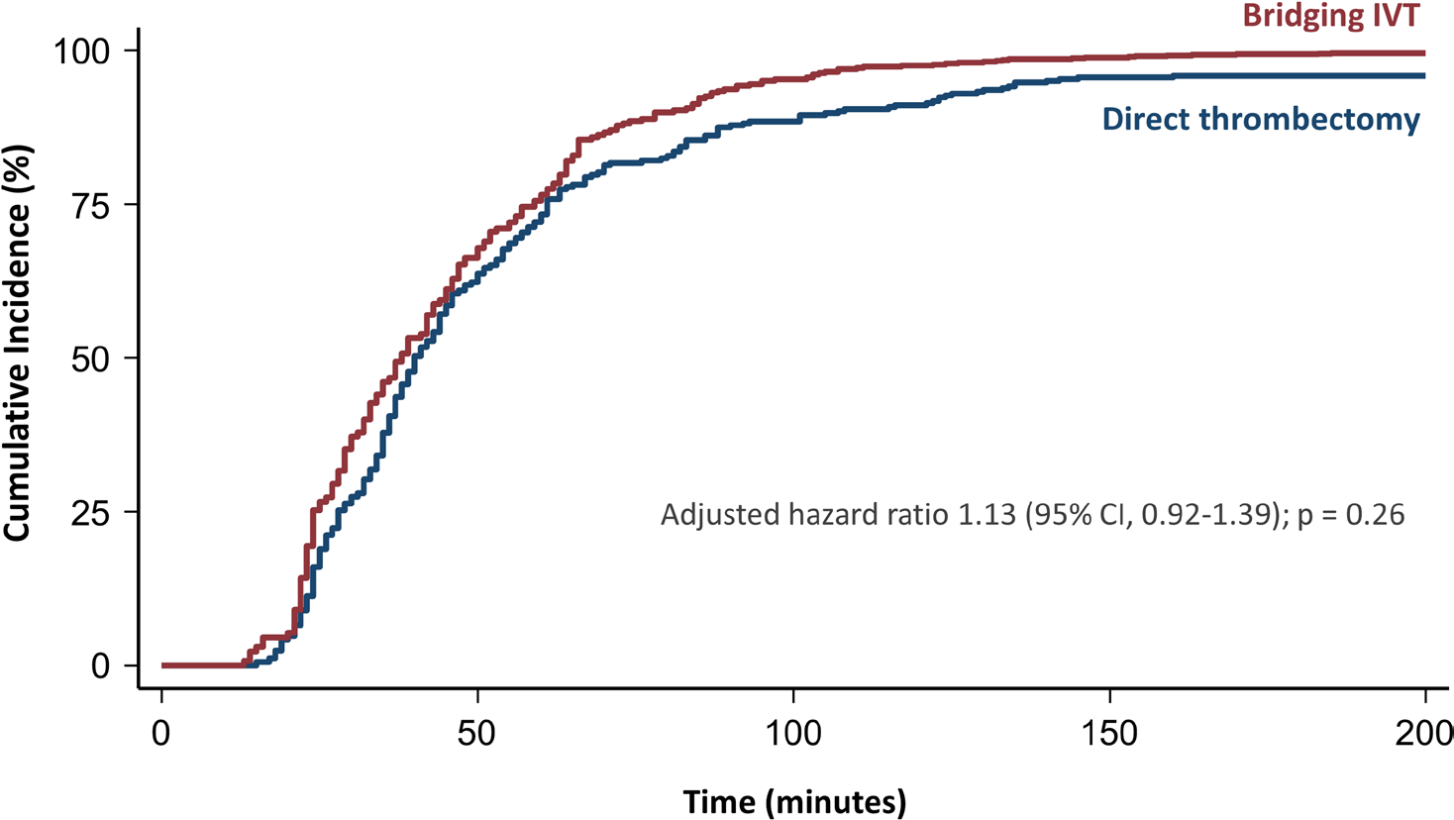
Time to reperfusion during thrombectomy in cardioembolic stroke. Covariate‐adjusted cumulative incidence functions for reperfusion in cardioembolic stroke. The displayed curves represent estimates derived from the multivariable Cox model adjusted for age, distal thrombus migration, National Institutes of Health Stroke Scale at baseline, occlusion site, tandem occlusion, carotid‐T occlusion, carotid stenting, Alberta Stroke Program Early CT Score, leptomeningeal collaterals, sedative regimen.

Successful reperfusion was achieved in 404 (86.3%) patients with cardioembolic stroke. Among patients with cardioembolic stroke, the effect of IVT on time to successful reperfusion varied depending on whether distal thrombus migration occurred following IVT and prior to thrombectomy. Patients who received both IVT and experienced distal thrombus migration exhibited a significantly lower hazard of achieving successful reperfusion at any given time point (aHR 0.57, 95% CI [0.34–0.96], *p* = 0.03). IVT alone was not associated with time to successful reperfusion (aHR 1.13, 95% CI [0.92–1.39], *p* = 0.26; Figure 3).

As expected, the presence of tandem occlusion was associated with a lower hazard of successful reperfusion (aHR 0.59, 95% CI [0.38–0.91], *p* = 0.018). Also, the presence of leptomeningeal collaterals was associated with a higher hazard of successful reperfusion (aHR 1.69, 95% CI [1.26–2.27], *p* < 0.001). A detailed description of the Cox proportional hazards model as well as validity assessments is provided in the Supporting Information (Table S10 and Information S4). The model was stratified by carotid T occlusion status to account for non‐proportional hazards related to this variable. The results of the test of proportional hazards are detailed in the Supporting Information (Table S11).

### 3.4 Effect of Intravenous Thrombolysis on the Number of Aspiration Attempts During Thrombectomy by Stroke Etiology

In patients with large‐artery atherosclerotic stroke, IVT was independently associated with a reduction of aspiration attempts by 36% (incidence risk ratio (IRR) = 0.64, 95% CI [0.50–0.84], *p* = 0.001). We observed a significant interaction between IVT and TOAST subtype of cardioembolic strokes (*p* = 0.003) where IVT showed no measurable effect on the number of aspiration attempts (combined IRR ≈ 1.00, 95% CI [0.82–1.22]). Results for the full model are detailed in the Supporting Information (Table S12).

### 3.5 Effect of Groin‐to‐Recanalization Time on 90‐Day Functional Outcome

A longer GTR time was independently associated with a shift toward worse functional outcome, whereby each additional 10 min of GTR time increased the odds of a worse mRS outcome by 8.6% (adjusted OR = 1.09, 95% CI [1.05–1.13], *p* < 0.001). The full model is displayed in the Supporting Information (Table S13).

## 4 Discussion

Our observations suggest that the impact of IVT on thrombectomy differs by stroke etiology in patients with acLVO. In non‐cardioembolic stroke, bridging IVT was associated with roughly a 40% higher likelihood of achieving successful reperfusion at any point during the procedure compared with direct thrombectomy. In contrast, in cardioembolic stroke, IVT did not alter the incidence of successful reperfusion during thrombectomy; however, when distal thrombus migration occurred after IVT, the likelihood of successful reperfusion at any time point was approximately 43% lower than with direct thrombectomy. Consistent with these findings, IVT‐treated patients with large‐artery atherosclerosis required about 36% fewer aspiration attempts to achieve recanalization, whereas no reduction in aspiration count was observed in cardioembolic strokes.

The effects of IVT in AIS patients undergoing thrombectomy is a matter of ongoing debate. A potential increase in hemorrhagic complications [12] is offset by a faster initiation of reperfusion therapy, which reduces ischemia time in salvaged tissue [13], and the possibility of early recanalization before thrombectomy. Yet, bridging IVT may cause the clot to migrate beyond the reach of thrombectomy, disperse into new vascular territories, or fragment into smaller emboli [13]. A retrospective cohort study of the International Stroke Perfusion Imaging Registry (INSPIRE) [14] and a meta‐analysis of functional outcomes in AIS patients who underwent IVT and thrombectomy versus thrombectomy alone observed a reduction in the number of thrombectomy passes required to achieve reperfusion in cases of bridging IVT before thrombectomy [15]. A per‐pass analysis of thrombi retrieved from 106 passes in 60 AIS patients treated with thrombectomy revealed no difference in the average number of passes with versus without IVT [16], although the analysis did not differentiate clot composition or stroke etiology and may have been underpowered. A multi‐center analysis of thrombi retrieved from 550 AIS patients who underwent thrombectomy with or without prior IVT also found no between‐group differences in the number of total passes and final mTICI [17]. However, as stated by the authors, different effects of IVT on, e.g., variations in clot composition were not considered and might have balanced each other out. The BRIDGE‐TNK trial found that AIS patients treated with tenecteplase prior to thrombectomy had a greater rate of successful reperfusion and a 9‐min decrease in the groin‐to‐recanalization time than those who underwent thrombectomy alone [18].

The effect of thrombectomy and IVT on reperfusion success rates and times may vary according to clot composition and origin. An analysis of the MR CLEAN Registry biobank in 332 AIS patients [19] and a prospective study in 105 AIS patients [20] indicated that cardioembolic thrombi contain a higher proportion of fibrin and platelets, whereas non‐cardioembolic thrombi were predominantly composed of RBCs. Fibrin‐rich thrombi have been associated with a greater number of recanalization attempts during thrombectomy which translate into lower reperfusion rates [8, 21, 22] while red blood cell (RBC)‐rich thrombi have been associated with facilitated reperfusion. This is proposed to be due to a higher friction and sliding resistance of fibrin‐rich thrombi [9, 23]. In line with that, an analysis of the multicenter RESTORE Registry of thrombotic material extracted via thrombectomy in AIS between 2018 and 2019, observed that clots retrieved in earlier passes had a higher RBC content whereas clots retrieved in later passes displayed a higher fibrin content [24]. Fibrin‐rich thrombi have also been suggested to be less sensitive to IVT compared to RBC‐rich thrombi [25, 26] in which fibrin is less densely packed allowing for higher infiltration of the lytic and increased thinning of the fibrin fibers [27] allowing faster and more successful reperfusion [28, 29, 30]. These observations are consistent with our finding of faster successful reperfusion during thrombectomy in patients with large artery atherosclerotic stroke, as thrombi of an atherosclerotic origin are considered to be RBC‐rich, while clots of cardiac origin are considered to be fibrin‐rich [16, 24, 31]. Logically consistent, the lack of benefit from bridging IVT in presumably cardioembolic clots in our study is in line with the reduced susceptibility of fibrin to IVT. However, a detrimental effect emerged specifically in the subgroup where distal thrombus migration occurred. In these patients, the likelihood of successful reperfusion was 43% lower compared to direct thrombectomy. While IVT fails to soften fibrin‐rich clots, it may promote their displacement into more distal, smaller‐caliber arterial segments. Such migration can increase technical difficulty and procedural duration, effectively turning a neutral treatment effect into a detrimental one for this specific subpopulation. However, an assessment of clots from 50 patients with acLVO retrieved via thrombectomy could not confirm a correlation between thrombus histopathology and stroke etiology or successful extraction [32]. Another study in thrombectomy patients even indicated that RBC composition was higher in cardioembolic stroke but the investigation was limited by a small sample size (*n* = 37) and it also showed an association between RBC‐rich clots and successful recanalization [33]. In our cohort, the likelihood of reperfusion at any given time point during thrombectomy after bridging IVT in patients with cardioembolic stroke was approximately 43% lower than in those who were treated with direct thrombectomy but this association was only apparent when distal clot migration occurred. In these patients, the clot may have traveled beyond the point in the artery where thrombectomy would still be effective, as highlighted by two recent negative randomized trials on thrombectomy for distal vessel occlusion [8, 34]. Beyond conventional red blood cell– versus fibrin‐rich histology, molecular analyses have identified neutrophil extracellular traps as key structural components of ischemic stroke thrombi that stabilize the fibrin network and may contribute to resistance to intravenous thrombolysis. This mechanism may be particularly relevant for understanding heterogeneous effects of IVT prior to thrombectomy, as partial clot destabilization without effective lysis could promote distal embolization or increase procedural complexity, warranting further investigation [35].

Strengths of our study include the well‐characterized prospective cohort of stroke survivors as well as the robustness of our statistical models. Generalizability may be limited because patients were included from a regional network registry. We only looked at the final grade of reperfusion and did not take into consideration the number of passes during thrombectomy. Histological characterization of the retrieved clots was not available. The consistency of the observed etiological differences across all multivariable models underscores that the impact of bridging thrombolysis on reperfusion speed is a robust finding, remaining independent of key clinical and procedural covariates such as baseline occlusion site, anesthesia strategy, and stroke severity. However, the observed association between bridging IVT and delayed reperfusion in cardioembolic stroke with distal thrombus migration should be interpreted cautiously, as the subgroup size was small, and residual confounding inherent to this observational study cannot be excluded despite robust statistical adjustments. We cannot rule that technological advances of angiography catheters during the observational periods have influenced thrombectomy performance in our cohort. We compared strokes of cardioembolic and non‐cardioembolic origin and observed distinct differences in the effect of bridging IVT on speed of thrombectomy. Yet, cryptogenic stroke is a highly heterogeneous group of unknown stroke etiologies, and even the condition of two possible known reasons for stroke is classified as cryptogenic. However, the results of our subgroup analysis in patients with large artery atherosclerotic stroke could reproduce the beneficial effect of IVT on time‐to‐recanalization observed in all non‐cardioembolic stroke patients, supporting the validity of our observations.

## 5 Conclusion

This registry analysis suggests that the effect of IVT on thrombectomy performance may differ by stroke etiology. Bridging IVT was associated with faster reperfusion in non‐cardioembolic acLVO, whereas no such benefit was observed in cardioembolic stroke, where distal clot migration was associated with delayed reperfusion. These associations should be interpreted cautiously and warrant confirmation in larger studies, but they may help inform future strategies aimed at identifying patients most likely to benefit from bridging thrombolysis.

## Author Contributions

**Annahita Sedghi:** data curation, formal analysis, investigation, methodology, project administration, visualization, writing – original draft. **Daniel P. O. Kaiser:** investigation, writing – review and editing, methodology. **Martin Arndt:** investigation, methodology, writing – review and editing. **Norma J. Diel:** investigation, writing – review and editing. Erik Simon: Investigation, writing – review and editing. **Witold H. Polanski:** investigation, writing – review and editing. **Volker Puetz:** conceptualization, supervision, resources, writing – review and editing. **Hagen B. Huttner:** supervision, resources, writing – review and editing. **Timo Siepmann:** conceptualization, methodology, project administration, resources, supervision, visualization, writing – review and editing.

## Funding

The authors have nothing to report.

## Conflicts of Interest

T.S. reports grants from Federal German Ministry of Health, Kurt Goldstein Institute and lecture fees from AstraZeneca and Dresden International University outside the submitted work. The other authors have nothing to disclose.

## Supporting information


**Data S1:** **Information S1:** Definitions of patient characteristics.
**Information S2:** Effect of bridging thrombolysis on time to reperfusion in non‐cardioembolic stroke.
**Information S3:** Effect of bridging thrombolysis on time‐to‐recanalization in atherosclerotic stroke.
**Information S4:** Effect of bridging thrombolysis on time to reperfusion in cardioembolic stroke.
**Table S1:** Baseline and study parameters with mode of acquisition.
**Table S2:** Demographic and baseline characteristics by stroke etiology.
**Table S3:** Missing data in the prospective registry of patients undergoing thrombectomy.
**Table S4:** Effect of bridging thrombolysis on time to reperfusion in non‐cardioembolic stroke; hazard ratios from the cox proportional hazards model.
**Table S5:** Effect of bridging thrombolysis on time to reperfusion in non‐cardioembolic stroke; test of proportional hazards assumption.
**Table S6:** Effect of bridging thrombolysis on time to reperfusion in non‐cardioembolic stroke excluding stroke of undetermined etiology; hazard ratios from the cox proportional hazards model.
**Table S7:** Effect of bridging thrombolysis on time to reperfusion in non‐cardioembolic stroke excluding stroke of undetermined etiology; test of proportional hazards assumption.
**Table S6:** Missing data in the prospective registry of patients undergoing thrombectomy.
**Table S7:** Effect of bridging thrombolysis on time to reperfusion in atherosclerotic stroke; test of proportional hazards assumption.
**Table S8:** Effect of bridging thrombolysis on time to reperfusion in cardioembolic stroke; hazard ratios from the cox proportional hazards model.
**Table S9:** Effect of bridging thrombolysis on time to reperfusion in cardioembolic stroke; test of proportional hazards assumption.
**Table S10:** Effect of bridging thrombolysis on number of aspirations during thrombectomy; parameter list of the negative binomial regression model.
**Table S11:** Association between groin to recanalization time and functional outcome; Parameter list of the ordinal logistic regression model.
**Figure S1:** Distribution of groin‐to‐recanalization times.
STROBE Checklist

## Data Availability

Deidentified, aggregate data will be made available upon reasonable request to qualified investigators with approval by the authors. Requests can be made to the corresponding author by email.
