# Supplementary material for: IV Thrombolysis Facilitates Interventional Reperfusion in Non‐Cardioembolic but Not Cardioembolic Stroke
Source: Ann Clin Transl Neurol. 2026 Mar 24;13(9):1893–902. doi: 10.1002/acn3.70370 (PMC13394534; doi:10.1002/acn3.70370)
Supplement: Supplementary file 1 [file ACN3-13-1893-s001.pdf]

IV Thrombolysis Facilitates Interventional Reperfusion in Non-Cardioembolic but not Cardioembolic Stroke

Supporting Information

Information S1

Definitions of Patient Characteristics and Adjudication

List of definitions

1. Occlusion sites

We included only patients with acLVO defined as complete occlusion of at least one large intracranial artery on computed tomographic angiography (CTA), i.e., the intracranial segment of the internal carotid artery (ICA), the M1 segment of the middle cerebral artery (MCA), the M2 segment of the MCA. This included combined occlusions, i.e., tandem occlusion, carotid T occlusion, carotid L occlusion, carotid I occlusion). We excluded patients with isolated anterior cerebral artery (ACA) occlusion because thrombectomy is less established in this territory. Posterior circulation occlusion was also excluded.

2. National Institutes of Health Stroke Scale at baseline

In community hospitals without neurological expertise, which serve as the spokes of our tele-stroke network, patients were assessed using the National Institutes of Health Stroke Scale (NIHSS) by a tele-stroke fellow at the coordinating tertiary stroke center via videoconference. Local neurologists assessed drip and ship patients who were transferred from a partner hospital with a neurology department. After transfer or direct admission to the tertiary stroke center, all patients were assessed by vascular neurologists. All patients were assessed for baseline NIHSS. In cases where the NIHSS score was not available but a detailed report of the neurological physical examination was available, the NIHSS score was determined post hoc by an investigator (AS). Mechanical ventilation with sedation was equivalent to a NIHSS score of 32 points.

3. Premorbid state

We considered patients to be dependent if regular assessment showed a continuing need for assistance in any dimension of activities of daily living [Supplementary ref. 1].

Supplementary Ref. 1 Katz S (1983) Assessing self-maintenance: activities of daily living mobility, and instrumental activities of daily living. J Am Geriatr Soc 31:721–727.

4. Chronic disease

- We defined a chronic disease as a condition that may affect functional independence. Diseases were classified according to the organ systems listed below, with one point awarded for each system:
- Cor/Vascular: coronary artery disease, history of myocardial infarction, high grade vitium, peripheral artery disease, non-compaction cardiomyopathy, history of carotid artery stenting
- Pulmonary: asthma, pulmonary hypertension, chronic obstructive pulmonary disease, chronic hypoventilation syndrome
- Renal: renal insufficiency
- Abdomen: chronic pancreatitis or hepatitis, sclerosing cholangitis, cholesterol crystal embolization, liver failure, previous Billroth I operation
- Other organ systems: neurodegenerative diseases, Churg-Strauss syndrome, rheumatoid arthritis, myasthenia gravis, hepatic steatosis, neurosarcoidosis, critical illness polyneuropathy, critical illness myopathy, polymyalgia rheumatica, systemic sclerosis, factor V Leiden, paranoid schizophrenia, infantile brain injury, muscular dystrophy, multiple sclerosis, morbid obesity

Adjudication

In this study, stroke etiology was determined as follows:

Initial Adjudication: Etiology was determined during the clinical routine at our tertiary stroke center based on the final medical reports. These reports represent a consensus finding reached during daily clinical rounds and were formally signed off by a senior stroke consultant. The classification followed the TOAST (Trial of Org 10172 in Acute Stroke Treatment) criteria, utilizing results from the complete diagnostic workup including 72h-ECG monitoring, echocardiography, vascular imaging (CT-angiography or MR-angiography) and cervical artery and transcranial sonography.

Data Entry and Quality Control: The data were prospectively entered into our registry. To ensure high data quality and consistency for this specific analysis, an investigator (AS) performed a secondary central review of all electronic health records and diagnostic findings to double-check the correctness of the assigned TOAST categories and procedural metrics.

Completeness of diagnostic work up and procedure quality: All patients underwent ECG, 72-hour ECG Monitoring and acute vascular imaging (CT-angiography or MR-angiography) and ultrasound except for 125 (15.7%) patients who deceased in the post-acute phase after admission. Additional echocardiography was performed in cases where etiology was not evident by atrial fibrillation of large artery stenosis. All interventions were performed at a high-volume, supra-regional certified tertiary stroke center. The procedures were carried out by a dedicated neuro-interventional team with extensive experience in mechanical thrombectomy, operating within a standardized 24/7 multidisciplinary framework. This institutional setting ensures a high degree of procedural consistency and minimizes variability related to operator experience, which supports the generalizability of our findings to other high-volume academic stroke centers.

Information S2

Effect of Bridging Thrombolysis on Time to Reperfusion in Non-Cardioembolic Stroke

The global Schoenfeld test indicated no significant violation (χ^2 = 8.21, degree of freedom, df = 8, p = 0.41). Although age showed borderline evidence of non-proportionality (p = 0.05), all other covariates met the assumption, supporting the overall validity of the model. The overall model fit was supported by a significant likelihood ratio test (χ^2 = 20.19, p = 0.001), indicating that the included covariates jointly contributed to explaining variation in time to successful reperfusion.

Information S3

Effect of Bridging Thrombolysis on Time-to-Recanalization in Atherosclerotic Stroke

Among patients with atherosclerotic stroke, IVT was independently associated with a shorter time to successful reperfusion. In the stratified Cox proportional hazards model, IVT was associated with an increased hazard of achieving successful reperfusion at any time point during thrombectomy (adjusted hazard ratio [aHR] 1.59, 95% CI[1.03, 2.45], p = 0.038). A detailed description of the Cox proportional hazards model is provided in Supplemental Table S6.

The proportional hazards assumption was assessed using Schoenfeld residuals. The global test indicated no significant violation (χ^2 = 4.74, degree of freedom, df = 7, p = 0.69). The overall model fit was supported by a marginally statistically significant likelihood ratio test (χ^2 = 13.70, p = 0.05),

indicating that the included covariates jointly contributed somewhat to explaining variation in time to successful reperfusion. Stratification by carotid stenting, sedative regimen and occlusion site was applied to account for potential non-proportional hazards in these variables. The global and individual test of proportional hazards for each covariate is reported in Table S7.

Information S4

Effect of Bridging Thrombolysis on Time to Reperfusion in Cardioembolic Stroke

The global Schoenfeld test indicated no significant violation ($\chi^2 = 12.51$, df = 10, $p = 0.252$). However, the subgroup of patients who received IVT and experienced distal thrombus migration showed moderate evidence of non-proportionality ($\chi^2 = 5.15$, df = 1, $p = 0.023$), warranting cautious interpretation of its effects over time. The model demonstrated good overall fit, as indicated by a highly significant likelihood ratio test ($\chi^2 = 33.28$, $p < 0.001$). This suggests that the covariates included in the model jointly contribute to explaining variation in the time to successful reperfusion.

Table S1

Baseline and Study Parameters with Mode of Acquisition

	**Available in prospective database**	**Extracted retrospectively via chart review**
Demographic characteristics	X	
Premorbid condition		X
Chronic disease		X
Arterial hypertension	X	X
Diabetes mellitus	X	
HbA1c		X
LDL-C		X
Stroke characteristics	X	
NIHSS at baseline	X	X
Carotid T occlusion		X
Tandem occlusion		X
Interventions	X	
Sedative regimen	X	
Procedural times, min	X	
Procedural outcomes	X	
mTICI	X	X

IQR, interquartile range; SD standard deviation; LDL, low density lipoprotein; HDL, high density IQR, interquartile range; SD, standard deviation; LDL, low-density lipoprotein; HDL, high-density lipoprotein; HbA1C, hemoglobin A1c; NIHSS, National Institutes of Health Stroke Scale; mRS, modified Rankin scale; ASPECTS, Alberta Stroke Program Early CT score; TOAST, Trial of Org 10172 in Acute Stroke Treatment; IVT, intravenous thrombolysis; mTICI, modified treatment in cerebral infarction score. Parameters of interest for our study that were not available in our registry were extracted by chart review by two independent investigators (AS, SS). In cases where catheter angiography was omitted, e.g. due to early recanalization or insufficient mismatch on perfusion imaging at the mother ship clinic after telemedicine consultation and subsequent drip-and-ship transfer, two experienced neuroradiologists (DK, AC) assessed the mTICI score post hoc using CT angiography as previously described [Supplementary ref. 3]. Consensus was reached for ambiguous findings.

Supplementary Ref. 2 Mair G, von Kummer R, Adami A, White PM, Adams ME, Yan B, Demchuk AM, Farrall AJ, Sellar RJ, Ramaswamy R, Mollison D, Boyd EV, Rodrigues MA, Samji K, Baird AJ, Cohen G, Sakka E, Palmer J, Perry D, Lindley R, Sandercock PA, Wardlaw JM, IST-3 Collaborative Group (2015) Observer reliability of CT angiography in the assessment of acute ischaemic stroke: data from the Third International Stroke Trial. Neuroradiology 57:1-9.

Table S2
Demographic and Baseline Characteristics by Stroke Etiology

Parameter	Non-Cardioembolic (n=330)	Cardioembolic (n=468)	p-value
Age (median [IQR])	69 [61, 80]	80 [73, 85]	0.00
Sex, female (n, %)	131, 39.70	282, 60.26	0.00
Premorbid independence (n, %)	71, 21.52	150, 32.05	0.001
Chronic disease (n, %)			0.01
Arterial hypertension (n, %)	281, 85.41	428, 91.65	0.01
Diabetes mellitus (n, %)	90, 27.27	136, 29.06	0.63
HbA1c (median [IQR])	5.8 [5.4, 6.3]	5.8 [5.4, 6.2]	0.58
LDL-Cholesterol (median [IQR])	2.6 [1.8, 3.2]	2.1 [1.7, 2.8]	0.00
NIHSS (median [IQR])	15 [10, 18]	16 [11, 19]	0.02
ASPECTS (median [IQR])	7 [6, 9]	8 [6, 9]	0.33
Occlusion site (n, %)			0.21
ICA intracranial	5	4	
L, M1 prox.-distal	276	376	
M1/2-transition, M2 prox.- distal	48	88	
Leptomeningeal collaterals on DSA (n, %)	276, 84.40	390, 83.33	0.38
Intravenous thrombolysis (n, %)	169, 51.21	226, 48.29	0.23
Sedative regimen (n, %)	226, 69.97	328, 71.00	0.81
Onset-to-needle (median [IQR])	108 [83, 139]	105 [80,135]	0.31
Needle-to-Groin (median [IQR])	128 [55, 158]	128 [65, 165]	0.47
Groin-to-recanalization (median [IQR])	58 [36, 99]	49 [32, 78]	0.002
Number of Aspirations (median [IQR])	2, [1,3]	2, [1,3]	0.96
mTICI (n, %)			0.26
0	34, 10.3	36, 7.7	
1	4, 1.2	4, 0.9	
2a	16, 4.8	24, 5.1	
2b	121, 36.7	149, 31.8	
2c, 3	155, 47.0	255, 54.5	
Successful reperfusion (mTICI > 2b)	276, 83.6	404, 86.3	0.41

IQR: interquartile range; NIHSS, National Institutes of Health Stroke Scale; mRS, modified Rankin scale; ASPECTS, Alberta Stroke Program Early CT score; DSA, digital subtraction angiography; TOAST, Trial of Org 10172 in Acute Stroke Treatment; IVT, intravenous thrombolysis; mTICI, modified treatment in cerebral infarction score.

Table S3

Missing data in the prospective registry of patients undergoing thrombectomy

Parameter	Missing data (n, %)
Age	0, 00
Sex, female	0, 00
Chronic disease	1, 0.13
Premorbid state	0, 00
Arterial hypertension	2, 0.25
Diabetes mellitus	0, 00
HbA1c	48, 6.02
LDL-Cholesterol	46, 5.76
ASPECTS	4, 0.50
Occlusion site	0, 0.00
Tandem occlusion	2, 0.25
Distal thrombus migration	4, 0.50
Leptomeningeal collaterals on DSA	3, 0.38
TOAST classification	3, 0.38
Intravenous thrombolysis	0, 00
Sedative regimen	13, 1.63
Onset-to-needle	6, 1.52
Needle-to-groin	5, 1.27
Groin-to-recanalization	76, 9.52
mTICI	4, 0.50

NIHSS, National Institutes of Health Stroke Scale; mRS, modified Rankin scale; ASPECTS, Alberta Stroke Program Early CT score; DSA, digital subtraction angiography; TOAST, Trial of Org 10172 in Acute Stroke Treatment; IVT, intravenous thrombolysis; mTICI, modified treatment in cerebral infarction score.

Table S4

Effect of Bridging Thrombolysis on Time to Reperfusion in Non-Cardioembolic Stroke

Hazard Ratios from the Cox Proportional Hazards Model

Parameter	Adjusted HR [95 % CI]	p-value
Age	0.99 [0.98-1.00]	0.07
NIHSS at baseline	1.01 [0.99-1.04]	0.20
Occlusion site	0.92 [0.67-1.26]	0.59
Tandem occlusion	0.70 [0.50-0.98]	0.04
Intravenous thrombolysis	1.40 [1.08-1.81]	0.01
Distal thrombus migration	2.51 [0.32-19.61]	0.38
Interaction: intravenous thrombolysis & distal thrombus migration	0.64 [0.08-5.29]	0.68

HR: hazard ratio; CI: confidence interval; NIHSS, National Institutes of Health Stroke Scale. Strata not shown (no HR available): Carotid stent, leptomeningeal collaterals, sedative regimen.

Table S5

Effect of Bridging Thrombolysis on Time to Reperfusion in Non-Cardioembolic Stroke

Test of Proportional Hazards Assumption

Parameter	χ^2	df	p-value
Age	3.99	1	0.05
NIHSS at baseline	0.04	1	0.84
Occlusion site	0.09	1	0.12
Tandem occlusion	0.07	1	0.24
Intravenous thrombolysis	0.10	1	0.75
Distal thrombus migration	0.43	1	0.51
Interaction: IVT & DTM	0.66	1	0.42
**Global**	8.21	8	0.41

χ^2: Chi-squared statistics; df: degree of freedom. NIHSS, National Institutes of Health Stroke Scale; IVT: intravenous thrombolysis; DTM: distal thrombus migration.

Table S6

Effect of Bridging Thrombolysis on Time to Reperfusion in Non-Cardioembolic Stroke Excluding Stroke of Undetermined Etiology

Hazard Ratios from the Cox Proportional Hazards Model

Parameter	Adjusted HR [95 % CI]	p-value
Carotid stent	1.98 [1.03-3.78]	0.04
NIHSS at baseline	1.00 [0.95-1.05]	0.96
Occlusion site	0.53 [0.20-1.41]	0.21
Tandem occlusion	0.54 [0.26-1.12]	0.10
Leptomeningeal collaterals	0.82 [0.32-2.15]	0.69
Intravenous thrombolysis	2.06 [1.06-3.98]	0.03
Distal thrombus migration	0.79 [0.06-10.19]	0.86
Interaction: intravenous thrombolysis & distal thrombus migration	4.86 [0.26-91.2]	0.29

HR: hazard ratio; CI: confidence interval; NIHSS, National Institutes of Health Stroke Scale. Strata not shown (no HR available): age, sedative regimen.

Table S7

Effect of Bridging Thrombolysis on Time to Reperfusion in Non-Cardioembolic Stroke Excluding Stroke of Undetermined Etiology

Test of Proportional Hazards Assumption

Parameter	χ^2	df	p-value
Carotid stent	3.18	1	0.07
NIHSS at baseline	1.68	1	0.20
Occlusion site	1.14	1	0.28
Tandem occlusion	0.07	1	0.79
Leptomeningeal collaterals	0.82	1	0.12
Intravenous thrombolysis	0.10	1	0.76
Distal thrombus migration	0.03	1	0.86
Interaction: IVT & DTM	0.22	1	0.64
**Global**	8.21	9	0.41

χ^2: Chi-squared statistics; df: degree of freedom. NIHSS, National Institutes of Health Stroke Scale; IVT: intravenous thrombolysis; DTM: distal thrombus migration.

Table S8

Effect of Bridging Thrombolysis on Time to Reperfusion in Atherosclerotic Stroke

Hazard Ratios from the Cox Proportional Hazards Model

Parameter	Adjusted HR [95 % CI]	p-value
Age	0.98 [0.97-1.00]	0.11
NIHSS at baseline	1.03 [1.00-1.07]	0.09
Leptomeningeal collaterals	1.20 [0.63-2.28]	0.57
Tandem occlusion	0.90 [0.38-1.67]	0.65
Carotid T occlusion	0.80 [0.38-1.67]	0.55
Intravenous thrombolysis	1.59 [1.02-2.45]	0.04
Distal thrombus migration	1.84 [0.88-4.16]	0.15

HR: hazard ratio; CI: confidence interval; NIHSS, National Institutes of Health Stroke Scale. Strata not shown (no HR available): Occlusion site, carotid stent, sedative regimen.

Table S9

Effect of Bridging Thrombolysis on Time to Reperfusion in Atherosclerotic Stroke

Test of Proportional Hazards Assumption

Parameter	χ^2	df	p-value
Age	0.01	1	0.91
NIHSS at baseline	2.05	1	0.15
Leptomeningeal collaterals	0.97	1	0.32
Tandem occlusion	0.00	1	0.95
Carotid T occlusion	0.27	1	0.60
Intravenous thrombolysis	0.23	1	0.63
Distal thrombus migration	1.16	1	0.28
**Global**	4.74	7	0.69

χ^2*: Chi-squared statistics; df: degree of freedom. NIHSS, National Institutes of Health Stroke Scale; IVT: intravenous thrombolysis; DTM: distal thrombus migration.*

Table S10

Effect of Bridging Thrombolysis on Time to Reperfusion in Cardioembolic Stroke

Hazard Ratios from the Cox Proportional Hazards Model

Parameter	Adjusted HR [95 % CI]	p-value
Age	1.00 [0.99-1.00]	0.42
NIHSS at baseline	0.99 [0.97-1.01]	0.40
Occlusion site	0.79 [0.61-1.02]	0.07
Tandem occlusion	0.59 [0.38-0.91]	0.02
Carotid stent	1.85 [0.58-5.94]	0.30
Leptomeningeal collaterals	1.69 [1.26-2.27]	0.00
Sedative regimen	0.72 [0.57-0.90]	0.004
Joint effect of IVT & DTM*		
DTM only	0.71 [1.00-5.17]	0.74
IVT without DTM	1.13 [0.92-1.39]	0.26
IVT with DTM	0.57 [0.34-0.96]	0.03

*HR: hazard ratio; CI: confidence interval. NIHSS, National Institutes of Health Stroke Scale; IVT: intravenous thrombolysis; DTM: distal thrombus migration. Strata not shown (no HR available): Carotid T occlusion. *Reference group: Patients who did not receive IVT and had no vessel change.*

Table S11

Effect of Bridging Thrombolysis on Time to Reperfusion in Cardioembolic Stroke

Test of Proportional Hazards Assumption

Parameter	χ^2	df	p-value
Age	0.06	1	0.81
NIHSS at baseline	0.99	1	0.31
Occlusion site	1.90	1	0.17
Tandem occlusion	0.91	1	0.34
Carotid stent	0.59	1	0.44
Leptomeningeal collaterals	0.01	1	0.94
Sedative regimen	2.80	1	0.10
Joint effect of IVT & DTM*			
DTM only	0.04	1	0.84
IVT without DTM	0.90	1	0.34
IVT with DTM	5.15	1	0.02
**Global**	12.51	10	0.25

χ^2: Chi-squared statistics; df: degree of freedom. NIHSS, National Institutes of Health Stroke Scale.
IVT: intravenous thrombolysis; DTM: distal thrombus migration.

Table S12

Effect of Bridging Thrombolysis on Number of Aspirations during Thrombectomy

Parameter list of the Negative Binomial Regression Model

Parameter	Raw coefficient	IRR	[95% CI]	p-value
Age	-0.01	0.99	[0.99-1.00]	0.001
ASPECTS	-0.02	0.98	[0.96-1.01]	0.24
Occlusion site	0.08	1.08	[0.94-1.24]	0.27
Tandem occlusion	0.32	1.37	[1.16-1.62]	< 0.001
Carotid T occlusion	0.22	1.25	[1.05-1.48]	0.01
Sedative regimen	0.16	1.18	[1.04-1.33]	0.01
Intravenous thrombolysis	-0.44	0.64	[0.50-0.84]	0.001
Intravenous thrombolysis # TOAST category 2	0.44	1.56	[1.16-2.09]	0.003

IRR: incidence rate ratio (raw regression coefficients were exponentiated to yield IRR); CI: confidence interval; ASPECTS: Alberta Stroke Program Early CT Score. TOAST category 2: TOAST, Trial of Org 10172 in Acute Stroke Treatment category 2 (i.e. cardioembolic stroke)

Table S13

Association between groin to recanalization time and functional outcome

Parameter list of the ordinal logistic regression model

Parameter	Odd ratio	[95% CI]	p-value
Age	1.05	[1.03-1.06]	< 0.001
Premorbid condition, needs assistance	3.95	[2.70-5.78]	< 0.001
NIHSS at admission	1.10	[1.10-1.13]	< 0.001
ASPECTS	0.91	[0.83-1.00]	0.05
Occlusion site	0.75	[0.51-1.11]	0.15
Tandem occlusion	1.37	[0.88-2.16]	0.17
Carotid T occlusion	1.24	[0.75-2.05]	0.41
Leptomeningeal collaterals	0.72	[0.48-1.07]	0.10
Sedative regimen	1.62	[1.15-2.26]	0.01
Intravenous thrombolysis	-0.44	[0.50-0.84]	0.001
All bleedings	2.46	[1.80-3.37]	0.001
HbA1c	1.37	[1.14-1.63]	0.001
LDL	0.91	[0.77-1.07]	0.26
HDL	0.63	[0.42-0.92]	0.02

CI: confidence interval; ASPECTS: Alberta Stroke Program Early CT Score. TOAST: Trial of Org 10172 in Acute Stroke Treatment; HbA1c: glycated hemoglobin; LDL: low-density lipoprotein; HDL: high-density lipoprotein.

Figure S1

Distribution of groin-to-recanalization times

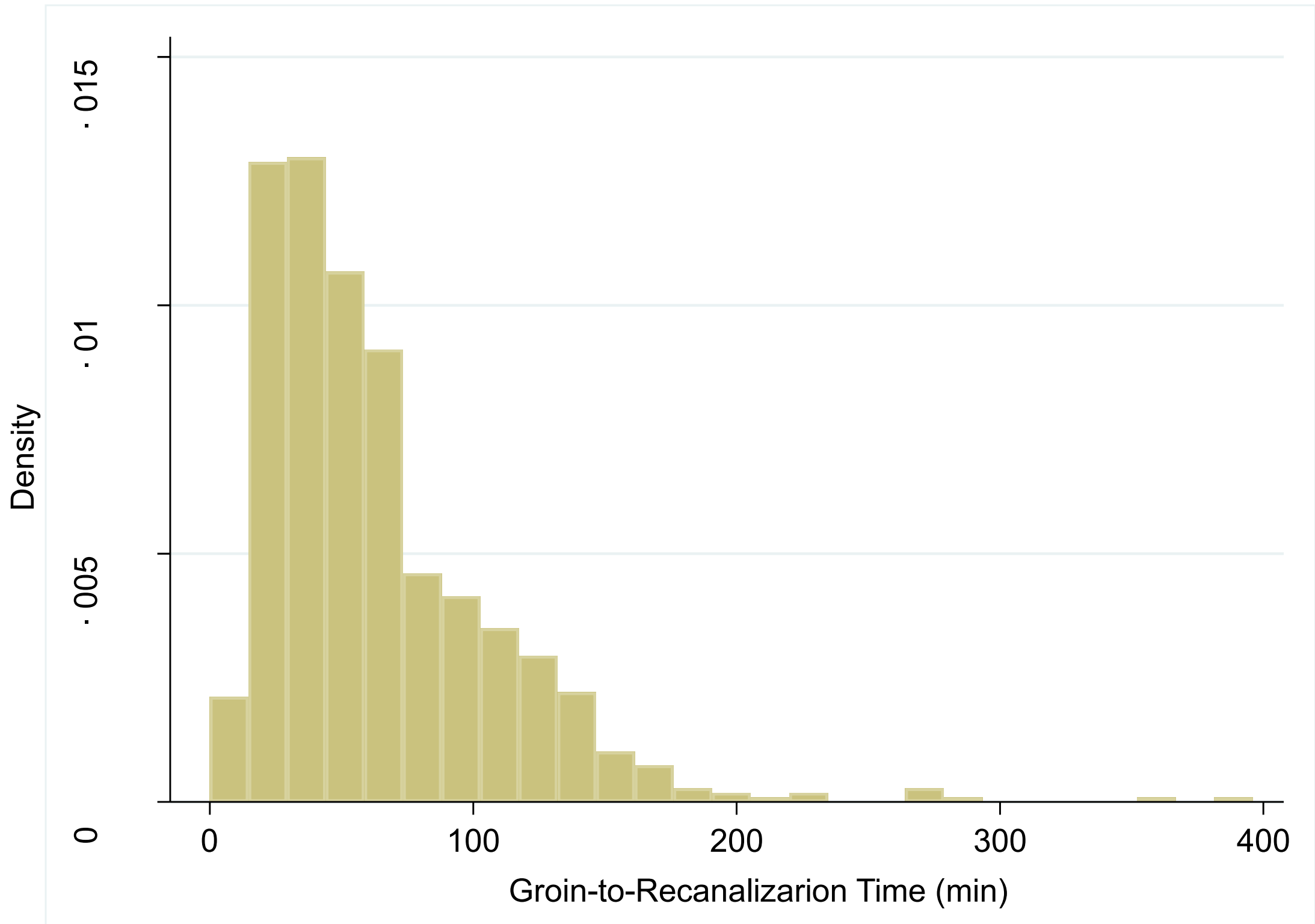

STROBE Checklist

	Item No.	Recommendation	Page No.	Relevant text from manuscript
**Title and abstract**	1	(*a*) Indicate the study's design with a commonly used term in the title or the abstract	2	"We performed a time-to-event analysis of consecutive patients with anterior circulation large vessel occlusion (acLVO) stroke from a prospective thrombectomy registry at a German tertiary stroke centre (January 2017-January 2023).
		(*b*) Provide in the abstract an informative and balanced summary of what was done and what was found	2	"IVT was associated with faster reperfusion during thrombectomy in non-cardioembolic acLVO, whereas in cardioembolic stroke with distal clot migration it was associated with delayed reperfusion."
**Introduction**				
Background/rationale	2	Explain the scientific background and rationale for the investigation being reported	3	"The authors emphasized the need for research to identify time- and person-specific factors that influence the effect of IVT among patients undergoing thrombectomy.7 Variations in the proportions of fibrin and red blood cell (RBC) content are exhibited by clots of patients with LVO, mainly between clots of cardiac and non-cardiac origin. These variations may influence the response to IVT before thrombectomy…"
Objectives	3	State specific objectives, including any prespecified hypotheses	3	"We aimed to assess the association of IVT with the time from groin puncture to recanalisation in patients with AIS who underwent thrombectomy for anterior circulation LVO (acLVO) depending on depending on whether the cause was cardioembolic or non-cardioembolic."
**Methods**				
Study design	4	Present key elements of study design early in the paper	4	"We conducted a post hoc analysis of our prospective registry of consecutive thrombectomy-eligible patients..."

Setting	5	Describe the setting, locations, and relevant dates, including periods of recruitment, exposure, follow-up, and data collection	4	"…consecutive thrombectomy-eligible patients treated from 01/01/2017 to 01/01/2023 at the tertiary stroke center of University Hospital Carl Gustav Carus in Dresden, Germany. Our thrombectomy registry includes both 'mothership' patients and those transferred from one of 13 hub-and-spoke telestroke network spokes or from one of eight partner primary stroke centers in eastern Saxony as previously described."
Participants	6	(*a*) *Cohort study*—Give the eligibility criteria, and the sources and methods of selection of participants. Describe methods of follow-up	4-6	"We included adults who received thrombectomy for acLVO in a time-to-event analysis. Eligible occlusion sites comprised the intracranial segment of the internal carotid artery or the M1 and/or M2 segment of the middle cerebral artery"<br>"Patients with good reperfusion and missing data on procedural times were excluded from the available case analysis."
		(*b*) *Cohort study* –<br>For matched studies, give matching criteria and number of exposed and unexposed<br>*Case-control study* –<br>For matched studies, give matching criteria and the number of controls per case	Figure 1	See Figure 1
Variables	7	Clearly define all outcomes, exposures, predictors, potential confounders, and effect modifiers. Give diagnostic criteria, if applicable	5-6	"The primary outcome was time from groin puncture to successful angiographic reperfusion…We constructed a multivariable stratified Cox regression model to assess the association between IVT and time to successful reperfusion. The model was adjusted for relevant clinical and procedural variables, including age, baseline stroke severity (NIHSS), presence of a tandem occlusion or carotid T occlusion, vessel occlusion site and occurrence of distal thrombus migration. To explore effect modification by stroke subtype, we conducted separate models for patients with cardioembolic stroke (Trial of Org 10172 in Acute Stroke Treatment, TOAST category 2)

				and those with non-cardioembolic etiologies (TOAST categories other than 2)."
Data sources/ measurement	8*	For each variable of interest, give sources of data and details of methods of assessment (measurement). Describe comparability of assessment methods if there is more than one group	4-5, Supporting information	"Parameters not available in our registry were extracted via chart review by an investigator (AS). Stroke etiology was determined according to TOAST criteria by senior stroke consultants during clinical routine and subsequently validated through a secondary central review of all medical records and diagnostic findings by a study investigator (AS). A list of parameters and their modes of acquisition is provided in the Supporting Information (Information S1, Table S1). "
Bias	9	Describe any efforts to address potential sources of bias	6	"We performed a sensitivity analysis, repeating the main analysis excluding patients with stroke of undetermined etiology from the non-cardioembolic stroke group. We performed a sub-analysis in patients with large atherosclerotic stroke among those with a non-cardioembolic etiology to exclude undetermined and other known causes of stroke using the same tests as in the main analysis."
Study size	10	Explain how the study size was arrived at	4, Figure 1	"We conducted a post hoc analysis of our prospective registry of consecutive thrombectomy-eligible patients treated from 01/01/2017 to 01/01/2023 at the tertiary stroke center of University Hospital Carl Gustav Carus in Dresden, Germany."

**Results**				
Participants	13*	(a) Report numbers of individuals at each stage of study - e.g. numbers potentially eligible, examined for eligibility, confirmed eligible, included in the study, completing follow-up, and analyzed	7, Figure 1	"We included 798 patients who underwent thrombectomy for AIS due to acLVO."
		(b) Give reasons for non-participation at each stage	Figure 1	See Figure 1
		(c) Consider use of a flow diagram	Figure 1	See Figure 1

Descriptive data	14*	(a) Give characteristics of study participants (e.g., demographic, clinical, social) and information on exposures and potential confounders	6-9, Table 1, Supporting information	"…(413 females [51.8%], median age 77 years [interquartile range, IQR 66-84 years], baseline NIHSS 15 [IQR, 10-19], median onset-to-recanalization time 302 minutes [IQR, 235-364], median groin-to-recanalization time 53 minutes [IQR, 34-86]). Of those, 395 [49.5%] received IVT prior to thrombectomy. All patients who received intravenous thrombolysis were treated with intravenous alteplase; tenecteplase and intra-arterial thrombolytics were not used during the study period. Among all patients, successful reperfusion was achieved in 680 patients [85.2%]. Cardioembolic stroke was the underlying cause of stroke in 468 patients [58.9%]. Among patients with non-cardioembolic stroke, distal thrombus migration occurred in 1/161 (0.6%) patients treated with direct thrombectomy and 14/169 (8.4%) patients receiving bridging IVT, while corresponding rates in cardioembolic stroke were 2/242 (0.8%) and 22/226 (9.7%), respectively (Table 1). Demographic data, vascular risk factors, and clinical and imaging characteristics were balanced between patients who underwent IVT before thrombectomy and those who were treated with direct thrombectomy (Table 1)."
		(b) Indicate number of participants with missing data for each variable of interest	8, Table S3	"The amount of missing registry data is low as detailed in Table S3."
		(c) *Cohort study* - Summarize follow-up time (e.g., average and total amount)	5, Figure 1	"In cases where procedural times were missing because of lack of any recanalization (mTICI 0 or thrombus not reached), we assigned a censoring time of 500 minutes to represent an upper limit of observed procedural duration as it exceeded the longest observed groin-to-recanalization time in our entire cohort (396 minutes) with buffer. The distribution of groin-to-recanalization times is shown the Supporting Information (Figure S1)."

Outcome data	15*	*Cohort study* - Report numbers of outcome events or summary measures over time	7-8, Table 1, Figure 2, Figure 3,	"Among all patients, successful reperfusion was achieved in 680 patients [85.2%]. Cardioembolic stroke was the underlying cause of stroke in 468 patients [58.9%]. Among patients with non-cardioembolic stroke, distal thrombus migration occurred in 1/161 (0.6%) patients treated with direct thrombectomy and 14/169 (8.4%) patients receiving bridging IVT, while corresponding rates in cardioembolic stroke were 2/242 (0.8%) and 22/226 (9.7%), respectively (Table 1)."
Main results	16	(*a*) Give unadjusted estimates and, if applicable, confounder-adjusted estimates and their precision (eg, 95% confidence interval). Make clear which confounders were adjusted for and why they were included	7-10, Supporting information	"Successful reperfusion was achieved in 276 (83.6%) patients with non-cardioembolic stroke. Among patients with non-cardioembolic stroke, IVT was independently associated with a shorter time to successful reperfusion (Fig 2)."
		(*b*) Report category boundaries when continuous variables were categorized	N/A	
		(*c*) If relevant, consider translating estimates of relative risk into absolute risk for a meaningful time period	N/A	
Other analyses	17	Report other analyses done - e.g. analyses of subgroups and interactions, and sensitivity analyses	9, Supporting information	"In the sensitivity analysis excluding patients with stroke of undetermined etiology from the group of non-cardioembolic stroke cases, IVT was associated with an increased hazard of achieving successful reperfusion at any time point during thrombectomy (aHR = 2.06, 95%CI [1.06-3.98], p = 0.033). The results of the proportional hazards test, as well as the global and individual proportional hazards tests for each covariate, are reported in the Supporting Information (Tables S6 and S7).<br>The sub-analysis in large artery atherosclerotic stroke also showed an independent association between bridging IVT and accelerated thrombectomy with an increased likelihood of successful reperfusion at any given time point during the procedure

				as detailed in the Supporting Information (Information S3 and Tables S8 and S9)."
**Discussion**				
Key results	18	Summarize key results with reference to study objectives	11	"Our observations suggest that the impact IVT on thrombectomy differs by stroke etiology in patients with acLVO. In non-cardioembolic stroke, bridging IVT was associated with roughly a 40% higher likelihood of achieving successful reperfusion at any point during the procedure compared with direct thrombectomy. In contrast, in cardioembolic stroke, IVT did not alter the incidence of successful reperfusion during thrombectomy; however, when distal thrombus migration occurred after IVT, the likelihood of successful reperfusion at any time point was approximately 43% lower than with direct thrombectomy. Consistent with these findings, IVT-treated patients with large-artery atherosclerosis required about 36 % fewer aspiration attempts to achieve recanalization, whereas no reduction in aspiration count was observed in cardioembolic strokes."
Limitations	19	Discuss limitations of the study, taking into account sources of potential bias or imprecision. Discuss both direction and magnitude of any potential bias	14	"Generalizability may be limited because patients were included from a regional network registry...However, the results of our subgroup analysis in patients with large artery atherosclerotic stroke could reproduce the beneficial effect of IVT on time-to-recanalization observed in all non-cardioembolic stroke patients, supporting the validity of our observations."
Interpretation	20	Give a cautious overall interpretation of results considering objectives, limitations, multiplicity of analyses, results from similar studies, and other relevant evidence	15	"This registry analysis suggests that the effect of IVT on thrombectomy performance may differ by stroke etiology. Bridging IVT was associated with faster reperfusion in non-cardioembolic acLVO, whereas no such benefit was observed in cardioembolic stroke, where distal clot migration was associated with delayed reperfusion. These associations should be interpreted cautiously and warrant confirmation in larger studies, but

				they may help inform future strategies aimed at identifying patients most likely to benefit from bridging thrombolysis.”
Generalizability	21	Discuss the generalizability (external validity) of the study results	14	“Generalizability may be limited because patients were included from a regional network registry.”
**Other information**				
Funding	22	Give the source of funding and the role of the funders for the present study and, if applicable, for the original study on which the present article is based	Declarations	See Declarations

*Give information separately for cases and controls in case-control studies and, if applicable, for exposed and unexposed groups in cohort and cross-sectional studies. **Note:** An Explanation and Elaboration article discusses each checklist item and gives methodological background and published examples of transparent reporting. The STROBE checklist is best used in conjunction with this article (freely available on the Web sites of PLoS Medicine at http://www.plosmedicine.org/, Annals of Internal Medicine at http://www.annals.org/, and Epidemiology at http://www.epidem.com/). Information on the STROBE Initiative is available at www.strobe-statement.org.
